# Chitosan‐protected dsRNA increases efficacy against *Helicoverpa armigera* without compromising biosafety for pollinators

**DOI:** 10.1002/ps.70837

**Published:** 2026-04-26

**Authors:** Daniel DN Vasquez, Lays A Teixeira, Rangel F Alves, Raire Cavalcante, Leonardo LP Macedo, Carmen SS Pires, Luciano P Silva, Maria CM Silva, Feras M Afifi, Carolina V Morgante, Martin G Edwards, Angharad MR Gatehouse, Maria F Grossi‐de‐Sa

**Affiliations:** ^1^ Embrapa Genetic Resources and Biotechnology Brasilia Brazil; ^2^ Catholic University of Brasília, Graduate Program in Genomic Sciences and Biotechnology Brasília Brazil; ^3^ Catholic University Dom Bosco, Graduate Program in Biotechnology Campo Grande Brazil; ^4^ National Institute of Science and Technology ‐ INCT PlantStress Biotech Brasília Brazil; ^5^ University of Brasilia Brasilia Brazil; ^6^ School of Natural and Environmental Sciences Newcastle University Newcastle upon Tyne UK

**Keywords:** biopesticide, cotton bollworm, nanoparticles, pollinators, risk assessment, RNA interference

## Abstract

**BACKGROUND:**

Cotton bollworm, *Helicoverpa armigera*, is a major global pest of cotton. Delivery of double‐stranded RNA (dsRNA) shows promise as a biopesticide for controlling this insect pest. However, employing RNA interference (RNAi) technology in the field requires enhancing its stability and demonstrating biosafety.

**RESULTS:**

Two genes, *chitin synthase II* and *cytochrome p450 protein 6b6*, were simultaneously targeted to disrupt the gut physiology of the cotton bollworm. Long dsRNA molecules (≈ 400 bp) encapsulated with a cationic polymer (chitosan) and a cross‐linker (tripolyphosphate, TPP) were synthesized to produce nanoparticles ranging from 150 to 300 nm in size, with a positive charge ~40 mV. Encapsulation resulted in a 100% increase in the knockdown effect on larvae that were fed with a diet containing dsRNA–nanoparticle complex compared to those fed with naked dsRNA. Additionally, nanoparticles produced substantially higher mortality than naked dsRNA, even at 100‐fold lower concentration. Overall, our findings suggest that chitosan/TPP/dsRNA nanoparticles at low doses (0.1–1 μg g^−1^) effectively enhance the RNAi effect in *H. armigera* larvae via oral delivery. In addition, acute oral toxicity tests were conducted on two relevant beneficial bees, *Scaptotrigona postica* and *Bombus terrestris*. Consistent with predictions from bioinformatic analyses, no significant lethality or signs of toxicity were observed in these non‐target organisms.

**CONCLUSION:**

Delivery of dsRNA molecules via nanoparticles was demonstrated to be a promising approach to enhance RNAi activity against *H. armigera*. Thus, this technology represents an efficient and safe strategy for managing lepidopteran pests. However, further research to optimize these molecules for field application is crucial. © 2026 The Author(s). *Pest Management Science* published by John Wiley & Sons Ltd on behalf of Society of Chemical Industry.

## Graphical abstract

Chitosan/tripolyphosphate/double‐stranded RNA (dsRNA) nanoparticles enhance RNA interference efficacy of orally delivered dsRNA against *Helicoverpa armigera*. Nanoparticles reduce the LC50 and do not cause deleterious effects or toxicity on beneficial insects. Nanoparticles at 1 μg g^−1^ caused more than 50% survival decrease while naked dsRNA was required at 100 μg g^−1^ to achieve similar mortality.

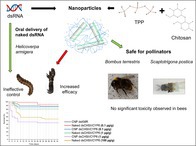

## 1 INTRODUCTION

The cotton bollworm (*Helicoverpa armigera*), a highly polyphagous and a major pest of maize, cotton, chickpea, tomato, sorghum, and soybean, among others, poses a significant threat to agriculture. Current estimates indicate that global losses in the agro‐industry due to this insect pest exceed $3 billion annually.^1^, ^2^ Despite the efficacy of synthetic pesticides and biopesticides, such as pyrethroids and *Bacillus thuringiensis* (*Bt*) Cry toxins, in reducing *H. armigera* populations to economically viable levels, the persistent emergence of resistance has raised concerns regarding the management of this pest species.^3^ Given the success of *Bt* technology, biotechnological approaches have garnered interest from companies aiming to develop products for insect pest management. For example, RNA interference (RNAi), a molecular mechanism for knocking down gene expression, has emerged as a promising tool for pest management, with two commercial products already approved: SmartStaxPro and Calantha.^4^, ^5^ The latter is a topically delivered double‐stranded RNA (dsRNA) designed to control the Colorado potato beetle (*Leptinotarsa decemlineata*); however, in lepidopteran insects like *H. armigera*, RNAi‐based approaches have shown lower efficacy,^6^ particularly when the dsRNA molecules are ingested by the larva.^7^, ^8^

Degradation in the gut and poor uptake of dsRNA molecules are considered the primary reasons for the inconsistency of RNAi in Lepidoptera. Consequently, the use of nanoformulations, a strategy to stabilize dsRNA and thus enhance RNAi efficacy in recalcitrant insects, has been widely explored in recent years.^9^ In two recent studies, chitosan (CS), a cationic polymer, demonstrated significant potential in boosting the RNAi response in *H. armigera* through the topical application of dsRNA‐loaded nanoparticles onto leaves ingested by the developing larvae.^10^, ^11^ Chitosan is a polysaccharide with low cytotoxicity that forms complexes with the negatively charged backbone of dsRNA via electrostatic interactions. In the presence of sodium tripolyphosphate (TPP), highly stable nanoparticles are formed, preventing the degradation of the nucleic acid in the insect gut lumen.^12^ Whilst the use of CS to protect dsRNA molecules has been well studied in a number of insect species,^13^, ^14^, ^15^, ^16^, ^17^ further optimization of the size of dsRNA molecules used and the properties of CS are still needed. Furthermore, safety assessments of nanoparticles and the dsRNA payload they contain will be required to commercialize this technology.

In this study, we examined the oral delivery of dsRNA/CS/TPP nanoparticles targeting *H. armigera*, utilizing longer dsRNA fragments (~400 bp) compared to those evaluated in previous studies. We selected target genes that were previously assessed in *H. armigera*, *chitin synthase II* (*CHSII*), *cytochrome p450 6B6* (*CYP6*), and *vacuolar ATP‐ase A* (*VATPa*), all of which are highly expressed in the gut and participate in different metabolic pathways essential for survival.^18^, ^19^ Additionally, we assessed the acute oral toxicity of the nanoparticles on two important pollinators, the native Brazilian stingless bee, *Scaptotrigona postica*, and the European buff‐tailed bumblebee (*Bombus terrestris*). We observed higher mortality in *H. armigera* when utilizing nanoparticle‐protected dsRNA compared to naked dsRNA, but no significant toxicity was found in non‐target organisms, even at very high doses of nanoparticle (10 μg/bee), thus demonstrating the biosafety of this approach.

## 2 MATERIALS AND METHODS

### 2.1 Insects

Eggs from *H. armigera* were obtained from a colony reared on an artificial diet provided by pragas.com (Piracicaba, SP, Brazil) and at 25 ± 2 °C, 60 ± 5% relative humidity (RH), and 12:12 h photoperiod. Workers of *S. postica* were obtained from colonies reared at Embrapa Genetic Resources and Biotechnology (Brasilia, DF, Brazil) under natural conditions. Individuals were collected for laboratory analysis during the toxicity tests and maintained at 28 ± 2 °C, 60 ± 5% RH, in full darkness. *B. terrestris* colonies were obtained from Koppert (Daventry, Northampton, UK), maintained at 27 ± 2 °C, 60 ± 5% RH, and full darkness, and fed with pollen provided by Koppert. Further information on diet composition to rear *H. armigera* and *S. postica* is provided in Supporting Information, Table S1.

### 2.2 Design and synthesis of dsRNA

GeneBank sequences of *CHSII* (KT004448.1), *CYP6* (PP163005.1), and *VATPa* (KP090287.1) from *H. armigera* were retrieved for dsRNA design. In an attempt to avoid silencing non‐target genes, the dsRNA sequences were designed and screened for potential off‐targets within the *H. armigera* transcriptome using siRNA‐Finder (si‐Fi) software.^20^ Transcripts from *H. armigera* genome assembly (GCF_030705265.1) were used as the reference database. The minimum size of predicted siRNAs was set at 18 nucleotides, and for off‐target analysis, two mismatches were tolerated. The same analysis was performed against the predicted transcriptome of *B. terrestris* (GCF_910591885.1). However, in the case of *S. postica*, it was not possible to perform an off‐target prediction due to the lack of sequence availability for this species.

Fragments of ≈200 and ≈400 bp of each gene were utilized as templates for dsRNA synthesis. DNA templates of matching sizes were chemically synthesized, cloned into a pCloneEZ‐NRS‐Blunt‐Amp by Epoch Life Science Inc. (Missouri City, TX, USA), and transfected into One Shot OmniMAX 2 T1R Chemically Competent *E. coli* (Thermo Fisher Scientific, MA, USA). The three target genes were cloned into a single vector; then, primers specific for the 5′‐ and 3′‐ends of each gene, containing the T7 promoter sequence (TAATACGACTCACTATAG) at the 5′‐end, were designed to produce DNA templates of single‐dsRNA molecules for each gene by PCR. *In vitro* synthesis was carried out using the MEGAscript T7 Transcription Kit (Thermo Fisher Scientific) following manufacturer's instructions. In addition, control dsRNA molecules of ≈400 bp targeting the microbial kanamycin resistance gene (dsKMR) were purchased from Genolution Inc (Seoul, South Korea). Control dsKMR was purified following the same protocol from the MEGAscript T7 Transcription Kit. Different sizes and concentrations of dsKMR were tested by microinjection prior to bioassays to preclude any potential effects of these parameters on target genes expression.

### 2.3 dsRNA target screening and size selection

Prior to assessing oral toxicity of RNA molecules, the synthesized dsRNAs were assessed by injecting 1 μg of naked dsRNA into the haemocoel of third‐instar *H. armigera* larvae using a 700 Series Hamilton syringe (10 μL) (Hamilton Company, NV, USA). Tested molecules included each individual dsRNA targeting *CHSII*, *CYP6*, and *VATPa*, as well as the following combinations: *CHSII/CYP6*, *CHSII/VATPa*, *VATPa/CYP6*, and *CHSII/CYP6/VATPa*. This was performed while maintaining the 1 μg dsRNA dose for each target gene. Treated insects (30 individuals per treatment) were maintained on fresh artificial diet for 10 days, as described above. Mortality was recorded daily, and the most effective dsRNA fragments were selected for nanoparticle delivery experiments.

### 2.4 Synthesis and characterization of nanoparticles

Nanoparticles were prepared by ionic gelation following the protocol described in our submitted patent WO/2020/007450.^21^ Low molecular weight CS with 85% of deacetylation degree (Sigma‐Aldrich, St. Louis, USA) was solubilized in acetic acid 1% (pH 5.5) and filtered using a syringe filter 25 mm × 0.22 μm. Chitosan microparticles were formed by slowly adding TPP (Sigma‐Aldrich, St. Louis, USA) (10 mg mL^−1^) to achieve a CS/TPP ratio of 1:1 (w/w) under vigorous stirring at 25 ± 2 °C, allowed to rest for 5 min at room temperature and centrifuged for 10 min at 5000 × *g*. The precipitate was washed three times in distilled water to remove free chitosan and/or TPP. The resulting solution was sonicated for 10 min with 30% amplitude. The dsRNA/CS/TPP nanoparticles (CNPs) were prepared containing 100 ng μL^−1^ of dsRNA at the following N (nitrogen):P (phosphate) molar proportions: 2:1, 5:1, 10:1, and 50:1. Size and charge of the CNPs were determined by dynamic light scattering (DLS) and zeta potential measurement, respectively, using a Malvern Zetasizer Nano ZS (Malvern Panalytical, Worcestershire, UK). Three measurements were taken per sample (from three independent biological samples) under the following parameters: 25 °C, material Refractory Index (RI) of 1.59, and dispersant (Milli‐Q ultrapure water) RI and viscosity of 1.33 and 0.887, respectively. Samples for the DLS analysis were diluted to 1 ng μL^−1^ of dsRNA.

In addition, atomic force microscopy (AFM) analysis was performed using two aliquots (1 μL each) of the formulation used in the bioassays (10:1 N:P ratio and 400‐bp dsRNA). Each nanoparticle suspension was deposited onto freshly cleaved muscovite mica substrates and allowed to air‐dry under protected ambient conditions. After complete drying, the samples were mounted onto a metallic sample holder using double‐sided adhesive tape and positioned on the microscope stage. AFM analyses were performed in ambient air at approximately 22 °C using an atomic force microscope SPM‐9600 (Shimadzu, Kyoto, Japan) equipped with a scanner capable of a maximum scan area of 125 × 125 μm. Imaging was conducted in dynamic (tapping) phase mode using a rectangular cantilever with a conical silicon tip, featuring a nominal spring constant of 10–130 N/m and a resonance frequency range of 204–497 kHz. The sweep frequency was set to 1 Hz. Surface topography images were acquired over 5 × 5 μm areas with a resolution of 512 × 512 pixels. The obtained images were processed by applying scan‐plane correction and individual particle segmentation using the manufacturer's dedicated offline analysis software (Shimadzu SPM‐9600).

### 2.5 Evaluation of CNP stability and dsRNA release

Haemolymph and gut fluid were extracted from third‐instar *H. armigera* larvae to assess the *in vitro* stability of CNPs. Haemolymph was extracted from three individuals by performing a small cut in the larvae prolegs and allowing them to bleed out on ice‐cold Parafilm prior to collection a 1.5‐mL tube containing 100 μM phenylthiourea, used to inhibit phenoloxidase activity. Gut juice extraction was carried out as previously described with minor modifications.^22^ In brief, five individuals were held with tweezers by the anterior part of the body, and gentle pressure was applied longitudinally to the larvae to stimulate peristaltic movements across the gut. After emesis, the gut juice was collected into a tube and centrifuged at 14 000 × *g* for 20 min at 4 °C, and the supernatant was transferred to a new tube. The protein concentration of the haemolymph and gut juice was determined fluorometrically using the Quibit Protein Assay Kit in a Qubit 4 Fluorometer (Invitrogen, MA, USA). Extracted haemolymph and gut fluids were diluted using 50 mM Tris–HCl buffer to achieve total protein concentrations of 500 ng μL^−1^ for *ex vivo* assays.

The *ex vivo* assay was performed using 500 ng of naked dsKMR (400 bp) and dsKMR complexed with CS/TPP at 5:1 and 10:1 N:P ratios with 3 μL of the haemolymph or gut fluids. The reaction volume was adjusted to 25 μL by adding 50 mM Tris–HCL buffer (pH was adjusted to 7.0 for haemolymph and 9.0 for gut fluid). Control treatments included the same mixtures without dsRNA or body fluids. The samples were incubated at 37 °C for 30 min. After incubation, samples were mixed with 6X Gel Loading Buffer (Thermo Fisher Scientific, MA, USA) and loaded onto a 1.5% agarose gel. Electrophoretic mobility shift assay was performed to determine the stability of the CNPs. Target dsRNA bands were visualized and photographed using a Gel Doc EZ Gel Documentation System (Bio‐Rad, CA, USA).

### 2.6 Nanoparticle delivery to *H. armigera*

First‐instar *H. armigera* larvae were challenged with CNPs using a 10:1 ratio (N:P), which was selected based on DLS results. The artificial diet was mixed with the nanoparticles at two different concentrations (0.1 and 1 μg of dsRNA/g of diet). Each larva was isolated in a plastic cage of 3 × 3 × 3 cm with 0.2 g of diet containing nanoparticles or control treatments. These control solutions included a dsRNA fragment of 400 bp targeting the kanamycin resistance gene from transposon Tn5 in *E. coli* HB101 (dsKMR) and naked (without nanoparticles) dsRNAs against the selected *H. armigera* genes at concentrations of 0.1, 1, and 100 μg g^−1^. For survival analysis, 90 larvae per treatment were used and mortality was recorded daily until adult emergence. Diet containing nanoparticles or control solutions was changed 72 and 144 h after the initial delivery, irrespective of the total amount of diet consumed. Larvae were fed with CNPs for 9 days. Thereafter, fresh control diet was provided *ad libitum*. Larval weight was measured at two key points after initial delivery, 4 days (early larval development) and 18 days (close to pupation). Time (days) to pupation was also recorded for each treatment.

Total RNA was extracted from treated larvae (see above) 72 h after delivery of diet containing nanoparticles. Three samples of approximately 100 mg of larvae (three to six individuals depending on larval size) per treatment were used to extract total RNA using TRIzol Reagent according to the manufacturer's instructions (Invitrogen). The integrity of the RNA samples was evaluated by agarose gel (1% w/v) electrophoresis and quantified using a NanoDrop‐2000 spectrophotometer (Thermo Fisher Scientific). The RNA samples were treated with DNase I (1 U μL^−1^) (Invitrogen), and first‐strand cDNA was synthesized from 1 μg of total RNA using random hexamer and anchored oligo dT primers included in the SensiFAST cDNA Synthesis Kit (Meridian Bioscience, Nottingham, UK). Reactions were performed according to the manufacturer's instructions.

### 2.7 Knockdown validation by RT‐qPCR

The Reverse Transcription Quantitative Polymerase Chain Reaction (RT‐qPCR) reaction mixture contained 10 μL of SensiFAST SYBR® No‐ROX mix (2x) (Meridian Bioscience), 2 μL of cDNA, 2.5 μL of each, forward and reverse primers, to a final concentration of 200 nM each, and nuclease‐free water for a total volume of 20 μL. RT‐qPCR reactions were performed on a CFX96 Touch Real‐Time PCR Detection System (Bio‐Rad) under the following conditions: initial denaturation at 95 °C for 15 min, followed by 40 cycles of 95 °C for 10 s, 60 °C for 20 s, and 72 °C for 30 s. Primer efficiency was calculated using the MINER software, and relative gene expression analysis was performed following the 2^−ΔΔCt^ method,^23^ using qbase+ software (Biogazelle, Gent, Belgium). Genes encoding ribosomal proteins 13 and 18 (*RPS13* and *RPS18*) were used as reference genes for relative expression analysis. Primers sequences for each gene are given in Supporting Information, Table S2.

### 2.8 Pollinator bioassays

To assess the toxicity of CNPs against non‐target beneficial insects, acute oral toxicity tests on two important pollinators were performed: *S. postica* and *B. terrestris*. Toxicity assays were performed according to OECD protocol No. 247: Bumblebee, Acute Oral Toxicity Test.^24^ Both bee species were challenged with combined dsRNAs targeting *H. armigera CHSII* and *CYP6B6* genes (dsCHSII/CYP6). These were delivered as naked dsRNA molecules and as CNPs (N:P = 10:1) using 0.1, 1 and 10 μg of dsRNA/bee.

Adult workers from at least three different colonies of *S. postica* were fed with 30% (w/v) sucrose solution, 50% honey (from *A. mellifera*), and 4% gelatin, containing either of the following treatments: CNPs, naked dsRNA, or control solutions. Imidacloprid at 10 ng μL^−1^ was used as a positive control. In total, 60 bees per treatment were evaluated, separated into 500‐mL plastic pots (10 bees per pot) (Supporting Information, Fig. S1). Bees were acclimatized to the artificial diet for 24 h prior to treatment. Then, 200 μL of diet per pot was provided for each treatment for 4 days, renewing the diet every day. Mortality was recorded 12, 24, 48, 72, and 96 h post‐delivery of dsRNA and control solutions.

Toxicity tests on *B. terrestris* were performed on adult workers fed with 50% (w/v) sucrose solution containing different treatments (as above). Esfenvalerate at 10 ng μL^−1^ was used as a positive control. Bees were acclimatized to the artificial diet for 24 h prior to treatment. Forty bees per treatment were separated into individual cages and fed with 100 μL of testing and control solutions using a plastic syringe (2 mL) (Supporting Information, Fig. S2). Then, 24 h after delivery of treatment solutions, sucrose solution was provided every day for 3 days. Mortality was recorded at 12, 24, 48, 72, and 96 h after delivery of dsRNA and control solutions.

### 2.9 Statistics

Results from relative gene expression analysis were evaluated for statistical differences among treatments using one‐way ANOVA with multiple comparisons (Tukey's HSD). Mortality from toxicity bioassays was evaluated by survival curves using the Kaplan–Meier model, and statistical differences were estimated using the log‐rank test. All analyses were performed using the SPSS Statistics 27 software (IBM, NY, USA).

## 3 RESULTS

### 3.1 dsRNA specificity

For each target gene, two dsRNA molecules of different size ranges (≈200 and ≈400 bp) were designed and synthesized. The exact sizes of the dsRNAs and the number of predicted siRNAs for each one are detailed in Table 1. Overall, longer dsRNA fragments were predicted to yield a higher number of effective siRNAs targeting their respective mRNA sequences. In addition, the dsRNAs and predicted siRNAs were not homologous to off‐target genes within the *H. armigera* genome (Supporting Information, Fig. S3). The predicted siRNAs from dsCHSII and dsCYP6 corresponded to a single sequence, while both sizes of dsVATPa presented hits against three different copies of VATPase A. No matches were identified in the genome of *B. terrestris* for any of the designed dsRNA sequences.

**Table 1:** dsRNA design and off‐target analysis

Fragment size (bp)	Target genes	Total siRNA hits	Efficient siRNA hits
210	*CHSII*	103	56
420	*CHSII*	235	115
207	*CYP6B6*	120	55
400	*CYP6B6*	265	136
210	*VATPase A* (1)	190	108
	*VATPase A* (2)	190	108
	*VATPase A* (3)	190	108
409	*VATPase A* (1)	389	203
	*VATPase A* (2)	389	203
	*VATPase A* (3)	389	203

*CHSII*, chitin synthase II; *CYP6B6*, cytochrome p450 6B6 family; *VATPase A*, vacuolar ATPase subunit A.

### 3.2 Microinjection of dsRNA causes gene knockdown and larval mortality

**Figure 1:**
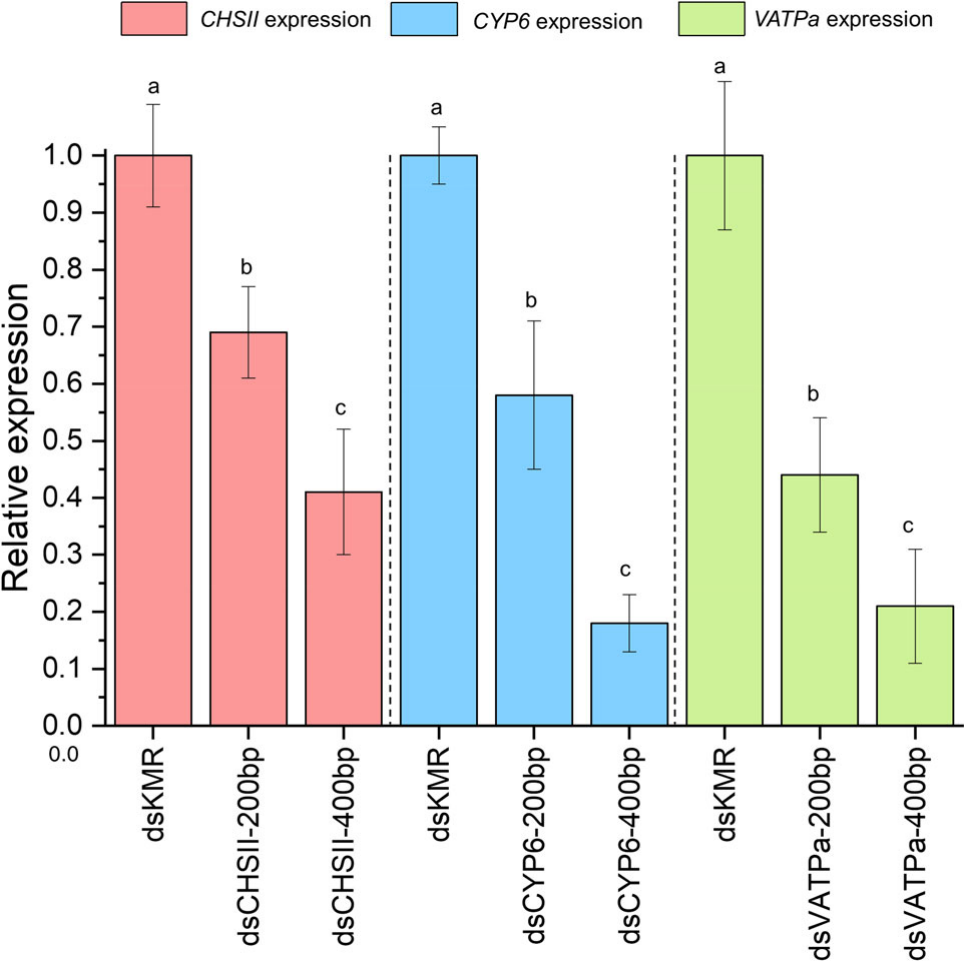
Injection of dsRNA molecules reduces the expression of corresponding target genes in *H. armigera*. Relative expression is presented as 2^−∆∆Ct^ (fold‐change values), using insects injected with dsKMR as a normalization factor set to 1. For each of the target genes both 200 and 400 bp fragments were used, whilst for dsKMR a 400 bp fragment was used. Lowercase letters denote significant differences at *P* < 0.05 (determined by one‐way ANOVA and Tukey's HSD). Error bars represent ±SE (*n* = 15). Gene expression analysis and statistical comparisons were performed for each gene individually, and the lack of differences in expression between different genes is depicted. Larvae were injected during the third‐instar stage. Samples were collected 48 h post‐injection. CHSII, chitin synthase II; CYP6, cytochrome P450 protein CYP6B6; VATPa, V‐ATPase subunit a; KMR, kanamycin resistance gene.

Gene expression knockdown induced by the dsRNA molecules, as a function of their size, was compared following the injection of naked dsRNAs into third‐instar larvae. Longer fragments (≈400 bp) induced a knockdown ranging from 1.5‐ to 3.5‐fold (compared to control dsKMR) higher than that of smaller fragments (≈200) for all target genes (Fig. 1). Therefore, the longer fragments of each dsRNA were selected for toxicity studies with *H. armigera*. Using these longer fragments, the mean transcript reduction of *CHSII* was 59.4% after 48 h, while for *CYP6* and *VATPa* gene expression was reduced by 82.3% and 78.9%, respectively. The use of dsKMR at a single concentration and size is supported by our data showing that neither concentration nor molecule size affected the expression of target genes (Supporting Information, Fig. S4).

**Figure 2:**
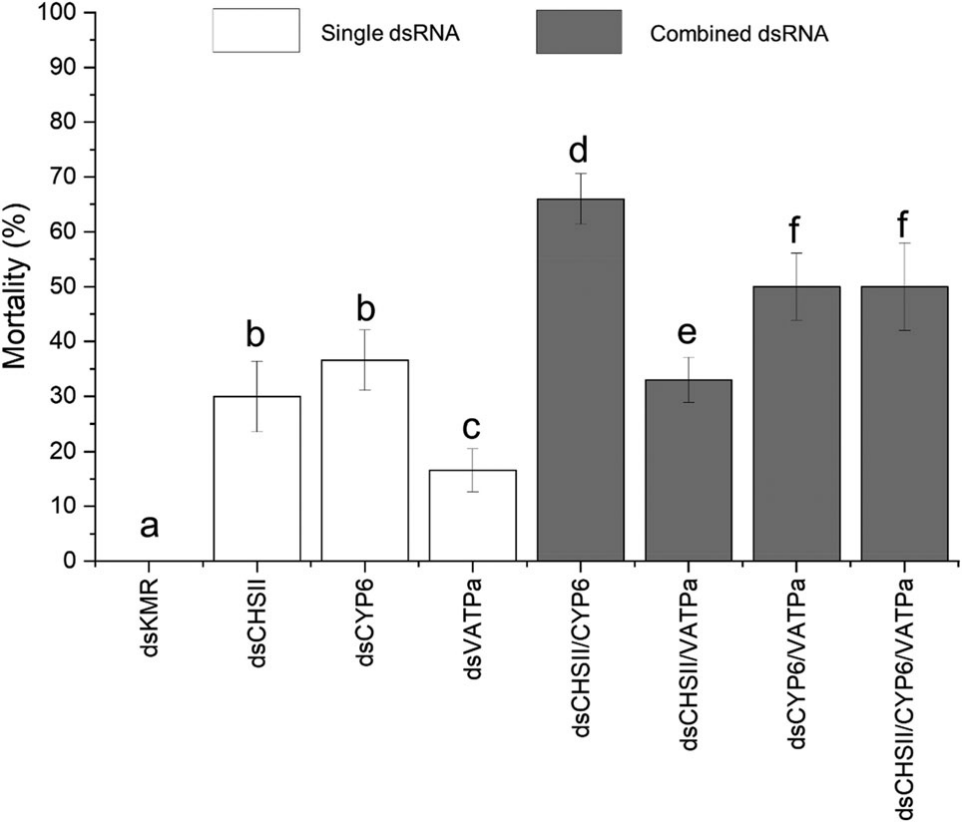
Injection of dsRNA combinations caused significantly greater mortality of third‐instar larvae. The graph illustrates the varying mortality effects of dsRNAs targeting single and combined genes post‐dsRNA injection. Lowercase letters denote significant differences at *P* < 0.05 (as determined by one‐way ANOVA and Tukey's HSD). Error bars represent ±SE (*n* = 30). Larvae were injected with 1 μg of dsRNA. *CHSII*, chitin synthase II; *CYP6*, cytochrome P450 protein CYP6B6; *VATPa*, V‐ATPase subunit a; *KMR*, kanamycin resistance gene.

Injection of a single dose of dsRNA (1 μg/larva), targeting *CHSII*, *CYP6*, and *VATPa* individually, caused 20.5%, 35.8%, and 16.9% mortality, respectively, after 10 days. However, when two or more dsRNAs were injected simultaneously (targeting more than one gene), significantly higher levels of mortality were achieved. Depending on the dsRNA combination, this ranged from 31.5% mortality when both CHSll and VATPa were simultaneously targeted, to 65.6% mortality when CHSll and CYP6 were simultaneously targeted. No mortality was observed in the control group where dsKMR was injected into the larvae (Fig. 2). As the most effective combination of dsRNAs in terms of *H. armigera* larval mortality was found to be dsCHSII/CYP6, this combination was selected for subsequent studies to evaluate whether use of nanoparticles increased subsequent toxicity when orally delivered.

### 3.3 Nanoparticle synthesis and characterization

To investigate whether the dsRNA size influences the physical characteristics of the chitosan nanoparticles, CNPs for both lengths of dsCHSII/dsCYP6 fragments were synthesized. An increase in dsRNA size led to an increase in nanoparticle size, with smaller fragments (≈200 bp) resulting in smaller particles measured by DLS, regardless of the N:P ratio utilized (Table 2 and Supporting Information, Fig. S5). However, dsRNA length was not the only factor affecting nanoparticle size. It was noted that at the highest N:P ratio, the size of nanoparticles increased significantly (exceeding 400 d.nm; Supporting Information, Fig. S5G,H). Interestingly, at smaller dsRNA lengths (Supporting Information, Fig. S5A,C,E), nanoparticle size did not increase up to N:P = 10:1, whilst for larger dsRNA (≈200 bp) differences sizes were observer among different N:P proportions, but no linear increase can be stated (Table 2).

**Table 2:** Physical parameters of different nanoparticle formulations measured by dynamic light scattering (DLS)

Parameter	Size (d.nm)		PDI		Zeta potential (mv)	
dsRNA size	200 bp	400 bp	200 bp	400 bp	200 bp	400 bp
N:P ratio						
2:1	164.1 ± 6.1^**a**^	232.8 ± 7.5^**b**^	0.43 ± 0.04^**ae**^	0.52 ± 0.04^**a**^	12.8 ± 0.2^**a**^	9.8 ± 0.6^**b**^
5:1	158.1 ± 3.6^**a**^	316.2 ± 3.3^**c**^	0.18 ± 0.01^**b**^	0.31 ± 0.01^**c**^	13.0 ± 0.2^**a**^	12.7 ± 0.1^**a**^
10:1	158.4 ± 9.1^**a**^	248.9 ± 4.7^**b**^	0.17 ± 0.01^**b**^	0.23 ± 0.01^**d**^	38.2 ± 0.7^**c**^	35.2 ± 1.0^**c**^
50:1	445.9 ± 8.3^**d**^	605.3 ± 11.8^**e**^	0.38 ± 0.01^**e**^	0.51 ± 0.01^**a**^	49.6 ± 0.8^**d**^	45.3 ± 0.9^**d**^

PDI, polydispersity index; DLS curves are presented in Fig. S5. Different superscript lowercase letters indicate significant differences among treatments for each parameter independently (Tukey test, *P* < 0.05, from three independent biological samples and three technical replicates).

The polydispersity of nanoparticles was also influenced by the N:P ratio, displaying higher values (>0.35) at both the lowest and highest ratios, and lower than 0.2 at intermediate N:P proportions for the smaller dsRNAs. In addition, longer dsRNA fragments showed higher polydispersity in all N:P proportions, excluding N:P = 2:1 (Table 2 and Supporting Information, Fig. S5).

Lastly, the zeta (*Z*) potential was not significantly altered in response to the dsRNA size beyond the 2:1 N:P ratio. Nevertheless, increasing this ratio enhanced the surface charge of the nanoparticles. Based on DLS analysis, nanoparticles generated with an N:P ratio of 10:1 and ≈400 bp dsRNAs were selected for the feeding bioassays because as they exhibited a notably positive charge (35.2 mV) and lower polydispersity compared to higher ratios (Table 2). Although formulations with a 200‐bp dsRNA at the same N:P proportion (10:1) presented slightly smaller polydispersity and size, we opted to use the longest dsRNA (≈400 bp) due to its stronger silencing effect in microinjection assays (Fig. 1). Data distribution for every parameter evaluated by DLS (size, polydispersity and *Z* potential) can be found in Supporting Information, Fig. S6A–C). Finally, AFM images obtained in dynamic (tapping) mode for nanoparticles with 10:1 N:P ratio and a 400‐bp length dsRNA (5 × 5 μm^2^) reveal a homogeneous distribution of chitosan nanoparticles over the substrate surface. Samples exhibit predominantly quasi‐spherical, well‐defined nanostructures with maximum heights reaching approximately 21.0 nm (D1) and 22.1 nm (D3), indicating similar vertical dimensions (Supporting Information, Fig. S7).

### 3.4 Nanoparticles protect dsRNA from nuclease activity

**Figure 3:**
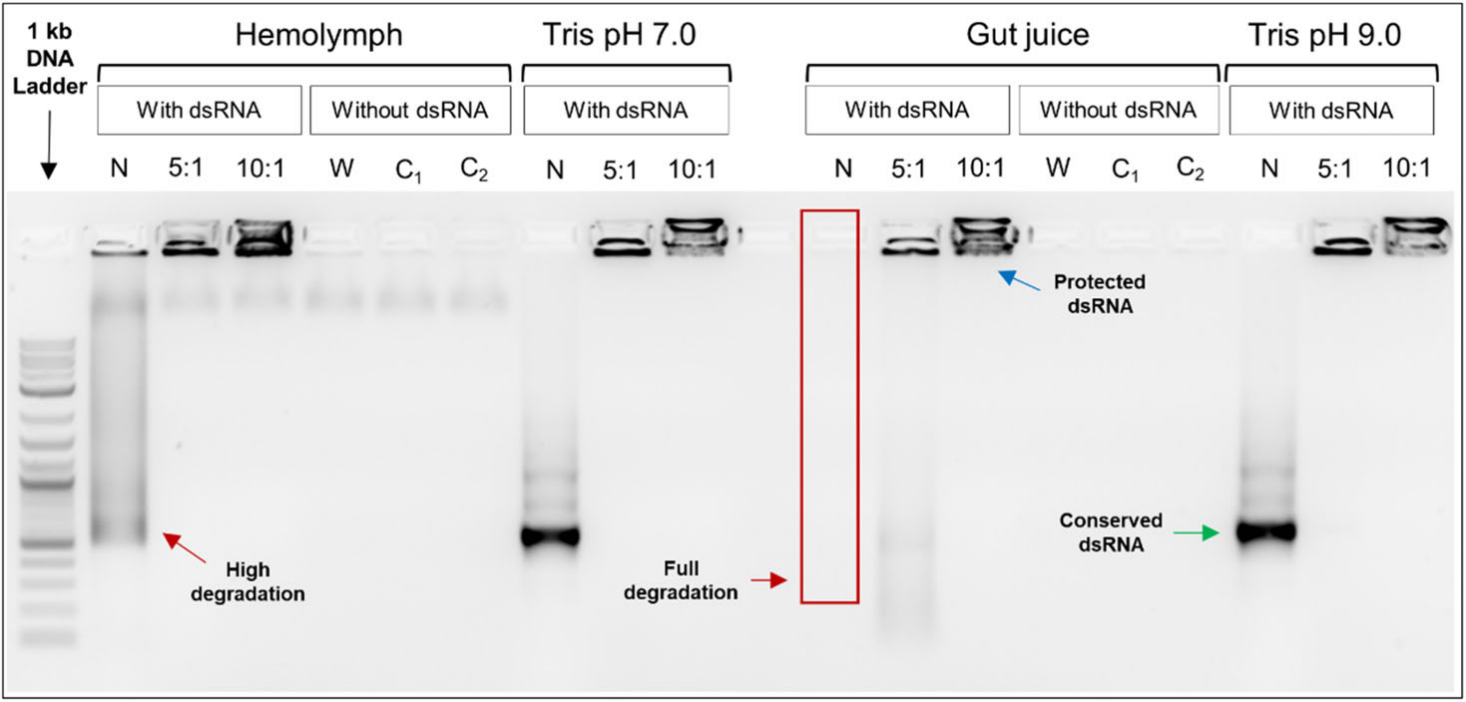
Naked dsRNA is completely degraded by gut fluids from *H. armigera* and partly degraded by the haemolymph *ex vivo*. Green arrow indicates a 400 bp fragment corresponding to intact dsRNA when incubated naked in Tris buffer. Red arrows show different levels of unprotected (N, naked dsRNA) dsRNA degradation (smear) when incubated in haemolymph and gut juice. An example of protected dsRNA is indicated by blue arrow, in which the RNA is retained in the well due to the size of CNPs (dsRNA/chitosan/TPP nanoparticles). No signs of degradation are observed for protected dsRNA. Ratios of N:P for CNPs are shown as 5:1 and 10:1. Sample without dsRNA or CS‐TPP are denoted by W. Control treatments without dsRNA consisted of insect fluid plus CS‐TPP nanoparticles at 5× (C1) and 10× (C2) CS concentrations relative to the naked dsRNA concentration. Control treatments with dsRNA included Tris buffer (50 mM) along with CNPs.

Naked dsRNA was completely degraded after a 30 min incubation with the gut juice of *H. armigera* larvae, and it was notably degraded, though not entirely, when incubated in haemolymph. In contrast, there was no sign of degradation observed in the dsRNA samples protected by CNPs when incubated with haemolymph. When exposed to gut juice, nanoparticles with a 5:1 N:P ratio exhibited a faint smear in the lower third of the gel, indicating slight degradation and a small release of dsRNA, whereas particles with a 10:1 N:P ratio showed no signs of degradation, with all nucleic acid remaining within the well of the agarose gel (Fig. 3).

### 3.5 CNPs enhanced toxicity of dsRNA to *H. armigera* by oral feeding

**Figure 4:**
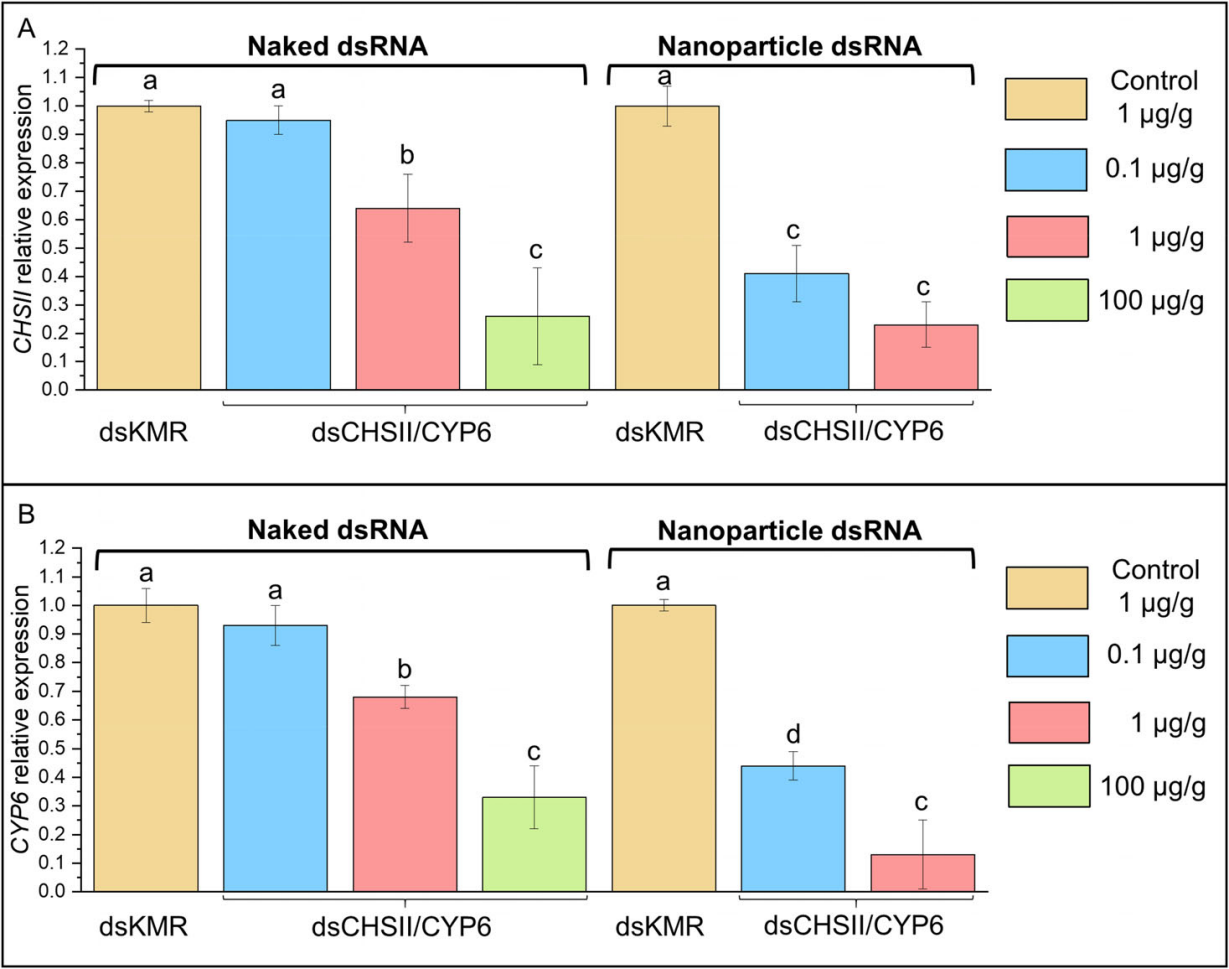
dsRNA nanoformulations knockdown expression of *H. armigera* target genes in a dose‐dependent manner when delivered orally: (A) *CHSII* expression and (B) *CYP6* expression. Relative expression is presented as 2^−∆∆Ct^ (fold‐change values), using the dsKMR sample as a normalization factor set of 1. Different lowercase letters denote significant differences at *P* < 0.05 (one‐way ANOVA and Tukey's HSD). Error bars indicate ±SE (*n* = 12). Dose is indicated as μg of dsRNA per g of diet. Samples were collected 72 h after diet delivering to neonate larvae. *CHSII*, chitin synthase II; *CYP6*, cytochrome P450 protein CYP6B6; *KMR*, kanamycin resistance gene; Naked, non‐encapsulated dsRNA.

Oral delivery of dsRNA encapsulated in CS:TPP nanoparticles significantly increased gene knockdown efficiency compared to naked dsRNA (Fig. 4). Interestingly, concentrations of dsRNA as low as 0.1 μg g^−1^ did not induce gene silencing unless protected by nanoparticles, while the delivery of 1 μg g^−1^ of protected dsRNA increased gene knockdown efficacy by approximately 5‐fold compared to dsKMR treatment. Furthermore, it was necessary to deliver naked dsRNA at concentrations 100 times greater (100 μg g^−1^) to achieve similar fold‐change values to those observed using encapsulated dsRNA. Transcript abundance of *CHSII* and *CYP6* was reduced up to 5‐ and 10‐fold, respectively, when utilizing protected dsRNA.

**Figure 5:**
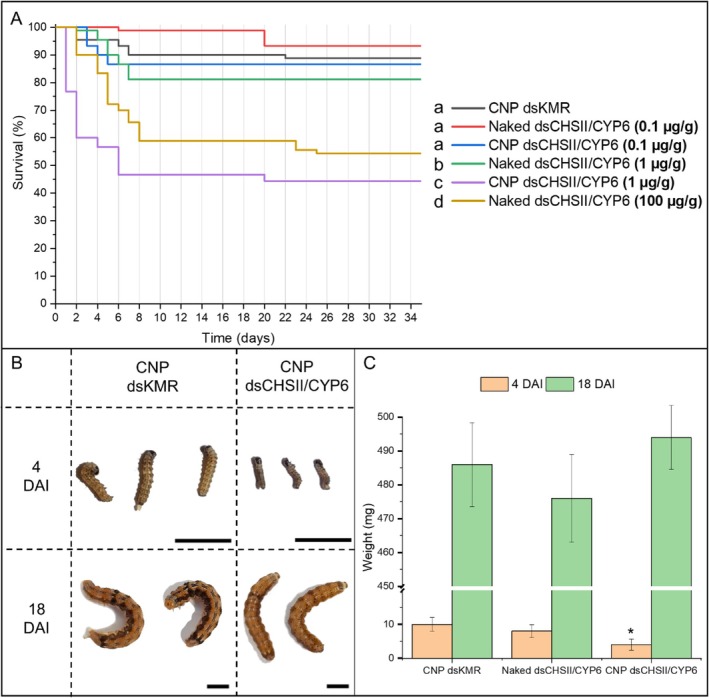
Nanoformulation of dsRNA significantly increases oral toxicity towards *H. armigera*. (A) Survival curves after 35 days of feeding on different concentrations of naked dsCHSII/CYP6 (0.1 μg g^−1^, 1 μg g^−1^, 100 μg g^−1^) or CNP dsCHSII/CYP6 (0.1 μg g^−1^, 1 μg g^−1^) compared to the control group (1 μg g^−1^). Different lowercase letters represent significant survival differences between the control group and the treatments (log‐rank test, *P* < 0.05, *n* = 90). (B) Morphological differences between the control group (CNP dsKMR at 1 μg g^−1^) and larvae fed with CNP dsCHSII/CYP6 at 1 μg g^−1^ after 4 and 18 days of nanoparticles delivery (DAI). (C) Weight of surviving larvae 4 and 18 DAI fed either control diet (CNP dsKMR at 1 μg g^−1^) or treatment diet (CNP dsCHSII/CYP6 at 1 μg g^−1^). Asterisk denotes significant differences between treatments and control CNP dsKMR, both at 1 μg g^−1^ (Dunnett's test, one‐way ANOVA *P* < 0.05). Scale bar: 5 mm. CHSII, chitin synthase II; CYP6, cytochrome P450 protein CYP6B6; KMR, kanamycin resistance gene; CNP, chitosan/TPP/dsRNA nanoparticles.

The knockdown of target genes in response to RNAi was further evidenced by the reduced survival of larvae treated with CNPs containing the combination of dsCHSII/CYP6 (Fig. 5). Protected dsRNA at 1 μg g^−1^ (highest concentration tested) was the only treatment to cause ≥50% mortality. In contrast, naked dsRNA at the same concentration resulted in less than 20% mortality, although at its highest concentration (100 μg g^−1^), naked dsRNA caused significant mortality (45%) when compared to the control treatment (dsKMR). At the lowest concentration (0.1 μg g^−1^), no survival differences were noted between larvae treated with dsKMR and those treated with dsCHSII/CYP6, irrespective of whether they were protected by the nanoformulation. In addition, survival declined more rapidly with CNP dsCHSII/CYP6 (1 μg g^−1^) compared to any other treatment, reaching ~50% in 6 days.

Interestingly, after 8 days, mortality rates decreased for all treatments (0.1 ± 0.1 deaths/day), and the survival curves leveled off until the end of the bioassay. Hence, up to day 8, the average mortality rate for CNP dsKMR and dsCHSII/CYP6 at 0.1 μg g^−1^ (both naked and NP) was 0.9 ± 0.3 deaths per day, while for dsCHSII/CYP6 at 1 μg g^−1^, the mortality rates was 2.1 (naked) and 6.0 (CNPs) deaths per day. Finally, during the same period, naked dsCHSII/CYP6 at the highest dose (100 μg g^−1^) exhibited a mortality rate of 3.9 deaths per day. Morphological signs of malnutrition, paralysis, and developmental defects were only observed during the early stages of the larval phase in individuals treated with CNP dsCHSII/CYP6 (Fig. 5(B)). In contrast, larvae treated with naked dsRNA (not shown) exhibited no differences to the dsKMR‐treated larvae (controls). In addition, the weight of surviving larvae treated with nanoparticle dsCHSII/CYP6 (4 ± 1.6 mg) differed significantly from the control treatment (dsKMR, 10 ± 2.1 mg) during the early developmental stages. However, by the fourth instar (18 days post dsRNA treatment) there were no significant differences in the mean larval weights among the different treatments (Fig. 5(C) and Supporting Information, Fig. S8). Nonetheless, a notable delay in pupation was observed in surviving larvae fed with nanoparticle dsCHSII/CYP6 (25–31 days), in contrast to those treated with dsKMR or naked dsCHSII/CYP6 (19–22 days). As expected, neither diet containing only water as an additive or CS/TPP nanoparticles without dsRNA caused any significant effects on mortality (Supporting Information, Fig. S9).

### 3.6 CNPs did not cause significant toxicity to insect pollinators

**Figure 6:**
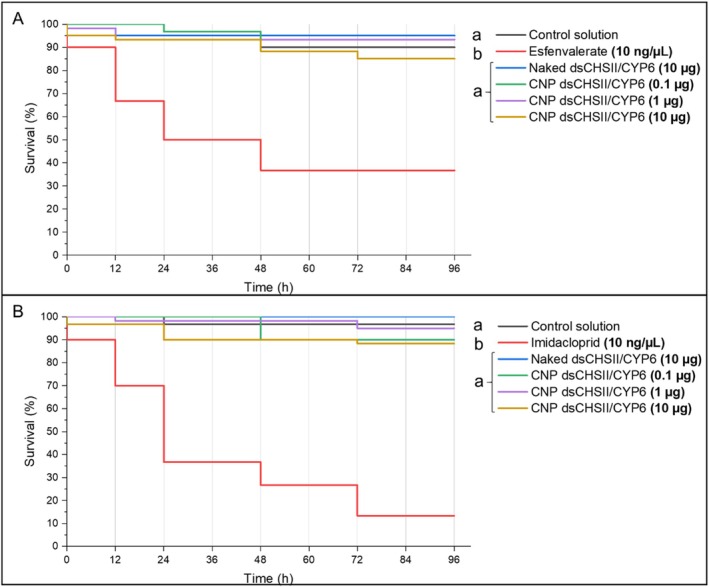
Neither naked nor CNP dsRNA was toxic towards two key pollinators in acute oral toxicity tests: (A) *B. terrestris* and (B) *S. postica*. Survival curves were estimated using the Kaplan–Meier model. Different concentrations of naked dsCHSII/CYP6 (0.1 μg g^−1^, 1 μg g^−1^, 100 μg g^−1^) or CNP dsCHSII/CYP6 (0.1 μg g^−1^, 1 μg g^−1^) were compared to the control group (1 μg g^−1^). Different lowercase letters represent significant survival differences between treatments (log‐rank test, *P* < 0.05). The experiment was performed on adult workers (*n* = 40 for *B. terrestris* and *n* = 60 for *S. postica*). Red curves represent the positive control (synthetic insecticide, esfenvalerate at 10 ng μL^−1^ for *B. terrestris*, imidacloprid at 10 ng uL^−1^ for *S. postica*). *CHSII*, chitin synthase II; *CYP6*, cytochrome P450 protein CYP6B6; *KMR*, kanamycin resistance gene; CNP, chitosan/TPP/dsRNA nanoparticles.

To confirm the specificity of the *in silico*‐designed dsCHSII/CYP6 and evaluate the potential toxicity of CNPs, bioassays were conducted testing encapsulated dsCHSII/CYP6 at different doses against two beneficial bee species. Compared to control diets (dsRNA‐free), neither naked nor CNP dsCHSII/CYP6‐treated bees exhibited any significant differences in survival, irrespective of the dose delivered (Fig. 6). Conversely, Esfenvalerate, a synthetic pyrethroid used as a positive control, resulted in high cumulative mortality for *B. terrestris* (63%). Similarly, imidacloprid caused 87% mortality in *S. postica*.

## 4 DISCUSSION

*H. armigera*, as reported for many other lepidopterans, exhibits lower efficacy in RNAi‐mediated silencing compared to insects such as *Drosophila* or *Diabrotica virgifera* from other orders, especially when dsRNA molecules are delivered orally.^6^ Whilst feeding dsRNA molecules to *H. armigera* can induce high levels of gene knockdown (up to 99%), the quantities of dsRNA required for this are far beyond feasible values for field applications.^7^ Some of the major reasons for this challenge are the degradation of dsRNA molecules within the lepidopteran gut, where the alkaline environment usually leads to increased nuclease activity and endosomal entrapment of dsRNA, blocking their path to the silencing complex.^8^ The utilization of nanoparticles for the sustainable management of *H. armigera*, either through use of nanocomposites working as the active ingredient itself^25^, ^26^, ^27^ or as carriers of other molecules,^28^, ^29^ has gained relevance in recent years. Chitosan polymers, in particular, have been employed to improve the stability and delivery of 21 bp siRNAs targeting *H. armigera* via foliar spray applications.^10^, ^11^ Another study used TPP as a crosslinker to augment chitosan nanoparticle stability in *Aedes aegypti*.^12^ In the present study, similar nanoformulations were evaluated for their capacity to enhance the stability and delivery of longer dsRNA molecules, aiming at enhancing the effectiveness of RNAi in *H. armigera*.

Long dsRNA molecules (≈400 bp) encapsulated by CNPs significantly increased the gene silencing effect in *H. armigera* compared to naked dsRNA when delivered orally. A low dose of 0.1 μg g^−1^ successfully triggered the knockdown of target genes in first‐instar larvae fed with 200 mg of diet containing dsRNA. Furthermore, we demonstrated that when the dsRNA was complexed with CS/TPP nanoparticles, it was markedly more effective for gene silencing than the 100 times more concentrated naked dsRNA (100 μg g^−1^). Similarly, Jaiwal *et al*. reported achieving around 60% gene knockdown using a dose of 8 μg per larva for the same species.^30^ In other studies, different approaches were utilized to achieve higher silencing using dsRNA or siRNA against *H. armigera*. The use of recombinant bacteria expressing dsRNA has shown promising results against *H. armigera*, with up to 90% knockdown.^19^, ^31^, ^32^, ^33^ However, the commercial viability of RNAi products based on recombinant bacteria faces several regulatory challenges.^34^ Likewise, the use of genetically modified (GM) plants expressing dsRNA or short‐hairpin RNAs for controlling *H. armigera* has been widely recognized as an effective approach from the onset of RNAi research in pest management.^18^, ^35^, ^36^, ^37^ Nevertheless, the shift toward non‐transformative and spray‐based strategies is viewed as a critical step in the development of new‐generation pesticides utilizing RNAi technology.^38^, ^39^

The size of the RNAi‐triggering molecule also needs to be considered. Longer dsRNA fragments resulted in stronger knockdown compared to shorter sequences for the three target genes evaluated. Currently, there is no consensus on whether the size of a given dsRNA positively or negatively affects the silencing efficacy. For instance, a compilation of RNAi experimental data by List *et al*. indicated that increasing the construct size might lead to reduced doses within a range of 100–600 bp.^40^ However, to date, there is a lack of studies in insects that definitively confirm a statistical correlation between increased dsRNA size and enhanced or diminished RNAi efficacy. In the case of *H. armigera*, using similar nanoformulations, we observed comparable knockdown levels with long ~400 bp dsRNA in this study compared to those reported by Kolge *et al*. using much shorter 21 bp siRNAs.^10^ Intriguingly, in a comparison of nuclear and transplastomic GM plants, Fu *et al*. revealed that silencing derived from siRNAs (produced by the plant RNAi machinery) was more effective and stable than that derived from dsRNA.^41^ This finding contrasts with the traditional understanding of RNAi in coleopteran species, where dsRNA fragments longer than 50 bp are typically required to effectively induce gene silencing.^42^ However, the presence of highly active dsRNA‐degrading enzymes and hostile pH conditions in the lepidopteran gut have been demonstrated to significantly reduce RNAi efficiency when dsRNA molecules are ingested.^43^, ^44^, ^45^ This scenario favours the delivery of siRNAs over longer fragments unless the longer molecules are protected by nanoformulations or other strategies to prevent degradation. In contrast, a study reported contradictory results, indicating that feeding *H. armigera* larvae with dsRNA molecules led to nearly a 3‐fold increase in silencing compared to feeding them with siRNAs.^30^ Therefore, it is imperative to conduct comprehensive studies comparing the degradation rates in the gut lumen and uptake efficiency by epithelial gut cells between siRNA and dsRNA when orally administered to lepidopteran species.

The RNAi improvement produced by our CNPs was also reflected in higher toxicity against *H. armigera* larvae when ingested. In earlier studies, substantial amounts (180 μg) of dsRNA were required to decrease larval survival by 40%^46^; other studies employing continuous feeding of dsRNA (20 μg/larva) over 10 days achieved similar mortality levels.^35^ In contrast, in the present study, amounts of less than 1 μg/larva were sufficient to achieve higher mortality (~55%). In some cases, the use of recombinant strategies has been more effective than direct delivery of dsRNA, producing mortality rates of around 80%.^31^, ^32^ Interestingly, the target genes employed in our nanoformulation, *CHSII* and *CYP6B6*, had previously been knocked down when the corresponding dsRNAs were delivered either via transgenic plants or recombinant bacteria targeting *H. armigera*.^18^, ^19^ Insect mortalities reported (up to 40%) in those studies were lower than those produced by CNPs used in the present study, and the effects were slower, only being observed in the pupal stage. Another notable observation was that most larvae treated with CNPs consistently exhibited delayed growth compared to control groups (Fig. 5 and Supporting Information, Fig. S8), indicating that the target genes in most of the larvae were silenced, at least to some degree. However, mortality achieved after 18 DAI was not 100%, and almost half of the larvae recovered and developed normally, suggesting a physiological recovery or variable knockdown efficacy among individuals.

Furthermore, our data demonstrated higher mortality rates when two or more target genes were simultaneously silenced, surpassing the impact of silencing single targets. The most effective dsRNA combination in our study specifically targeted two genes (*CHSII* and *CYP6*), whilst targeting three genes (*CHSII*, *VATPa*, and *CYP6*) exhibited slightly lower efficacy. Thus, we found no evidence that the increased mortality resulting from combining dsRNAs is linked to the number of targets themselves. Rather, we believe that the precise combination of dsRNAs capable of disrupting essential physiological processes synergistically presents an intriguing alternative to targeting single genes, particularly in efforts to delay the development of resistant populations. Resistance mechanisms to RNAi described to date are mainly associated with the processing machinery of dsRNA and cell import proteins; however, other mechanisms may also play a role. This could happen at the gene level and at the physiological level whereby the insect responds by overexpressing other copies of the gene, or even other genes that encode proteins that perform the same function as the silenced gene.^22^, ^47^, ^48^ For example, resistance in Colorado potato beetle was associated with lack of target gene knockdown and cross‐resistance to another dsRNA targeting the V‐ATPase subunit A gene.^49^ Targeting multiple genes will likely make it more difficult for the insect to respond, as simultaneous mutations would have to occur at different genome regions. Furthermore, it could be energetically prohibitable for viability, as the insect would have to respond to separate metabolic pathways.

A critical aspect for advancing topical RNAi‐based pesticides involving the use of nanoparticles is the assessment of the biosafety of these nanoformulations. Chitosan is recognized as an environmentally friendly biopolymer with diverse agricultural applications.^50^ It is categorized as a Minimum Risk Pesticide by the US government and has received approval for agricultural use by the EU.^51^, ^52^ Nevertheless, nanoparticles containing chitosan are affected by several factors and synthesis parameters that can alter their size, charge, shape, and other properties.^53^ This variability makes it necessary to employ rigorous risk assessments to evaluate the toxicity of CS nanoparticles as a prerequisite for the commercialization of this technology. Whilst risk assessment studies in the medical and pharmaceutical fields are established,^54^, ^55^ they are less well explored for agricultural applications, where it is paramount that sensitive ecosystems comprising closely related arthropods are not disrupted. Studies by Kolge and colleagues evaluated the specificity of CS/TPP/siRNA nanoparticles by testing their toxicity against two non‐target species, *Drosophila melanogaster* and *Spodoptera litura*, reporting no significant toxicity.^10^, ^11^ In addition to assessing effects on non‐target species, it is essential to include toxicity tests on beneficial and highly sensitive insects, such as bees, in these risk assessments. In this study, we demonstrated that at the highest dose (10 μg/bee), no significant mortality or signs of toxicity were observed for two species of pollinator, *S. postica* and *B. terrestris*, 96 h post‐ingestion of the CS/TPP/dsRNA nanoparticles. Whilst the biosafety studies presented here were limited to effects on mortality, they do provide a good starting point. Future studies should be extended to investigate long‐term effects, such as those on fecundity and behaviour. Furthermore, the effects on the transcriptional profiles of homologous genes, gut microbiota, and contact toxicity should also be determined to better evaluate the biosafety of the technology. Indeed, some studies have reported that chitosan nanoparticles, while biodegradable in nature, exhibit toxicity levels dependent on the applied concentration.^56^, ^57^

## 5 CONCLUSION

In this study, we demonstrated that oral delivery of CS/TPP nanoparticles encapsulating dsRNA is effective as previously reported for CS/TPP/siRNA nanoparticles in enhancing gene silencing in the devastating polyphagous insect pest *H. armigera*. Furthermore, we investigated different lengths of dsRNA molecules and their impact on the essential characteristics of the nanoparticles, revealing how payload size directly influences RNAi efficacy. For the first time, we also assessed the acute oral toxicity of CS/TPP nanoparticles on economically vital pollinators from Brazil (*S. postica*) and Europe (*B. terrestris*), demonstrating no harm to these beneficial bee species, highlighting the biosafety of using RNAi‐based strategies. Thus, the application of CS/TPP nanoparticles for stable and safe dsRNA delivery marks a groundbreaking advancement in RNAi‐based insect pest management. This innovative approach not only enhances the efficacy of insect pest control but also holds the promise of transforming agricultural practices into more sustainable and environmentally friendly solutions. With further optimization, commercially viable sprayable biopesticides that fully harness the potential of RNA interference technology could be developed, contributing to a new era in insect pest control that protects crops and supports ecosystems.

## CONFLICT OF INTEREST

The authors declare that the research was conducted in the absence of any commercial or financial relationships that could be construed as a potential conflict of interest.

## Supporting information


**Figure S1.** Materials used for toxicity bioassays with *Scaptotrigona postica*. (A) A Falcon tube (50 mL) lid containing vermiculite and water was used to ensure that the humidity was sufficiently high. (B) An Eppendorf (1.5 mL) lid was used as a feeder for the delivery of diet containing dsRNA. (C) Bioassays were carried out in plastic pots (500 mL). (D) *S. postica* adult worker.
**Figure S2.** Materials used for toxicity bioassays with *Bombus terrestris*. (A) Cage used for maintaining the *B. terrestris* colony. Bioassays were carried out using small cages (B), each of which was attached to a 2‐mL syringe containing testing solutions (C).
**Figure S3.** Predicted siRNAs produced by our *in silico* designed dsRNAs against *H. armigera*: (A) dsCHSII 420 bp, (B) dsCHSII 210 bp, (C) dsCYP6 400 bp, (D) dsCHCYP6 207 bp, (E) dsVATPa 409 bp, (F) dsVATPa 210 bp. The algorithm assumes that Dicer can cleave the dsRNA molecule at any point. Each position presented in the *x* axis indicates the possible start of an siRNA. Thus, the *Y* axis indicates how many siRNAs derived from *x* starting point in the dsRNA sequence (*x*‐mer) will have a hit in the mRNA target sequence given the parameters chosen.
**Figure S4.** Expression of *Ha*CHSII (top) and *Ha*CYP6 (bottom) after injection of control dsRNA molecules (dsKMR) with different sizes and amounts. Relative expression is presented as 2^−∆∆Ct^ (fold‐change values), with average expression as normalization factor. No statistical differences were observed under *P* < 0.05 (determined by one‐way ANOVA). Error bars represent ±SE (*n* = 9). Larvae were injected during the third‐instar stage. Samples were collected 48 h post‐injection. CHSII, chitin synthase II; CYP6, cytochrome P450 protein CYP6B6; KMR, kanamycin resistance gene.
**Figure S5.** Dynamic light scattering (DLS) analysis of dsRNA/CS/TPP nanoparticles: (A) N:P = 2:1, 200 bp; (B) N:P = 2:1, 400 bp; (C) N:P = 5:1, 200 bp; (D) N:P = 5:1, 400 bp; (E) N:P = 10:1, 200 bp; (F) N:P = 10:1, 400 bp; (G) N:P = 50:1, 200 bp; (H) N:P = 50:1, 400 bp. N:P indicates the phosphorous:nitrogen ratio of the nanoparticle. Size of dsRNA fragment is given in bp.
**Figure S6.** Distribution of physicochemical parameters of chitosan/TPP/dsRNA nanoparticles measured by dynamic scattering light analysis: (A) size, (B) zeta potential, (C) polydispersity. The *x* axis indicates the N:P ratio (phosphorous:nitrogen) of the nanoparticle. The size of dsRNA fragment is given in red (200 bp) and blue (400 bp) legends.
**Figure S7.** Nanoparticles with 10:1 N:P ratio and a 400‐bp length dsRNA visualized by atomic force microscopy (AFM) analysis. Imaging was conducted in dynamic (tapping) phase mode using a rectangular cantilever with a conical silicon tip, featuring a nominal spring constant of 10–130 N/m and a resonance frequency range of 204–497 kHz. The sweep frequency was set to 1 Hz. Surface topography images were acquired over 5 × 5 μm areas with a resolution of 512 × 512 pixels.
**Figure S8.** Weight distribution of larvae fed with dsRNA molecules (encapsulated, NP; non‐encapsulated, naked). Red boxes indicate data from larvae at 4 DAI (A) and blue boxes data from larvae at 18 DAI (B). KMR: kanamycin resistance gene (mock control). CHSII, chitin synthase II; CYP6, cytochrome P450 6B6; DAI, days after ingestion of dsRNA.
**Figure S9.** *Helicoverpa armigera* larval survival after ingestion of diet containing different molecules. Neonate larvae were fed with diet containing (1 μg g^−1^) of naked dsRNA, CNPs, water or CS/TPP nanoparticles. Cumulative mortality after 35 days after ingestion was compared among treatments. Different lowercase letters indicate significant differences at *P* < 0.05 (as determined by one‐way ANOVA and Tukey's HSD). CHSII, chitin synthase II; CYP6, cytochrome P450 protein CYP6B6; KMR, kanamycin resistance gene; CNP, chitosan/TPP/dsRNA nanoparticles; CS/TPP, nanoparticles of chitosan and TPP without dsRNA.
**Table S1.** Artificial diet composition for *H. armigera* and *S. postica*.
**Table S2.** Primers used for RT‐qPCR analyses. Melting temperature for all primers is 62 °C.

## Data Availability

The data that supports the findings of this study are available in the supplementary material of this article.
