# Supplementary material for: Chitosan‐protected dsRNA increases efficacy against Helicoverpa armigera without compromising biosafety for pollinators
Source: Pest Manag Sci. 2026 Apr 26;82(8):7746–57. doi: 10.1002/ps.70837 (PMC13352321; doi:10.1002/ps.70837)
Supplement: Supplementary file 1 — Figure S1. Materials used for toxicity bioassays with Scaptotrigona postica. (A) A Falcon tube (50 mL) lid containing vermiculite and water was used to ensure that the humidity was sufficiently high. (B) An Eppendorf (1.5 mL) lid was used as a feeder for the delivery of diet containing dsRNA. (C) Bioassays were carried out in plastic pots (500 mL). (D) S. postica adult worker. Figure S2. Materials used for toxicity bioassays with Bombus terrestris. (A) Cage used for maintaining the B. terrestris colony. Bioassays were carried out using small cages (B), each of which was attached to a 2‐mL syringe containing testing solutions (C). Figure S3. Predicted siRNAs produced by our in silico designed dsRNAs against H. armigera: (A) dsCHSII 420 bp, (B) dsCHSII 210 bp, (C) dsCYP6 400 bp, (D) dsCHCYP6 207 bp, (E) dsVATPa 409 bp, (F) dsVATPa 210 bp. The algorithm assumes that Dicer can cleave the dsRNA molecule at any point. Each position presented in the x axis indicates the possible start of an siRNA. Thus, the Y axis indicates how many siRNAs derived from x starting point in the dsRNA sequence (x‐mer) will have a hit in the mRNA target sequence given the parameters chosen. Figure S4. Expression of HaCHSII (top) and HaCYP6 (bottom) after injection of control dsRNA molecules (dsKMR) with different sizes and amounts. Relative expression is presented as 2−∆∆Ct (fold‐change values), with average expression as normalization factor. No statistical differences were observed under P < 0.05 (determined by one‐way ANOVA). Error bars represent ±SE (n = 9). Larvae were injected during the third‐instar stage. Samples were collected 48 h post‐injection. CHSII, chitin synthase II; CYP6, cytochrome P450 protein CYP6B6; KMR, kanamycin resistance gene. Figure S5. Dynamic light scattering (DLS) analysis of dsRNA/CS/TPP nanoparticles: (A) N:P = 2:1, 200 bp; (B) N:P = 2:1, 400 bp; (C) N:P = 5:1, 200 bp; (D) N:P = 5:1, 400 bp; (E) N:P = 10:1, 200 bp; (F) N:P = 10:1, 400 bp; (G) N:P = 50:1, 200 bp; [file PS-82-7746-s001.docx]

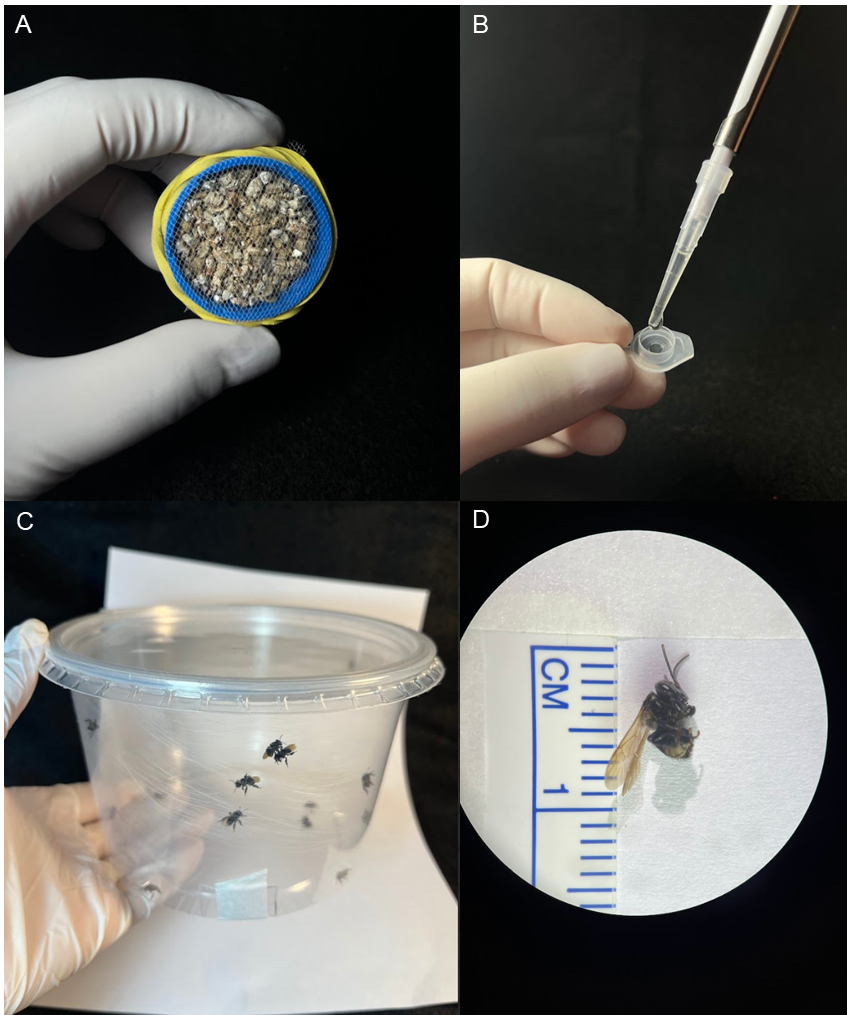


**Fig. S1**. Materials used for toxicity bioassays with *Scaptotrigona postica*. A) Falcon tube (50 mL) lid containing vermiculite and water was used to ensure that the humidity was sufficiently high. B) Eppendorf (1.5 mL) lid was used as a feeder for the delivery of diet containing dsRNA. C) Bioassays were carried out in plastic pots (500 mL). D) *S. postica* adult worker.


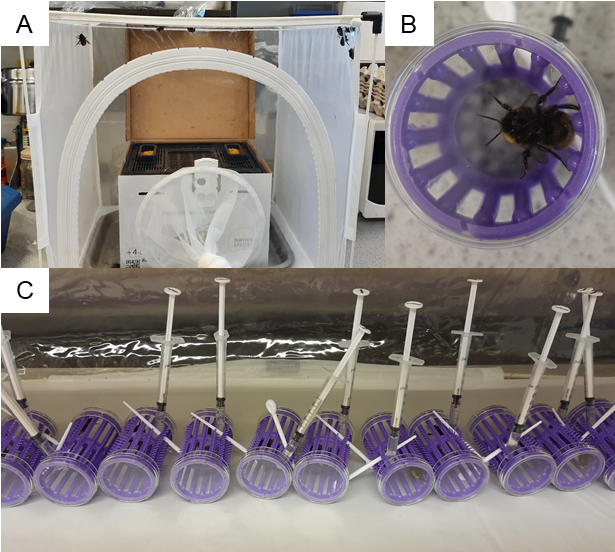


**Fig. S2**. Materials used for toxicity bioassays with *Bombus terrestris*. A) Cage used for maintaining the *B. terrestris* colony. Bioassays were carried using small cages (B), each of which was attached to a 2 mL-syringe containing testing solutions (C).


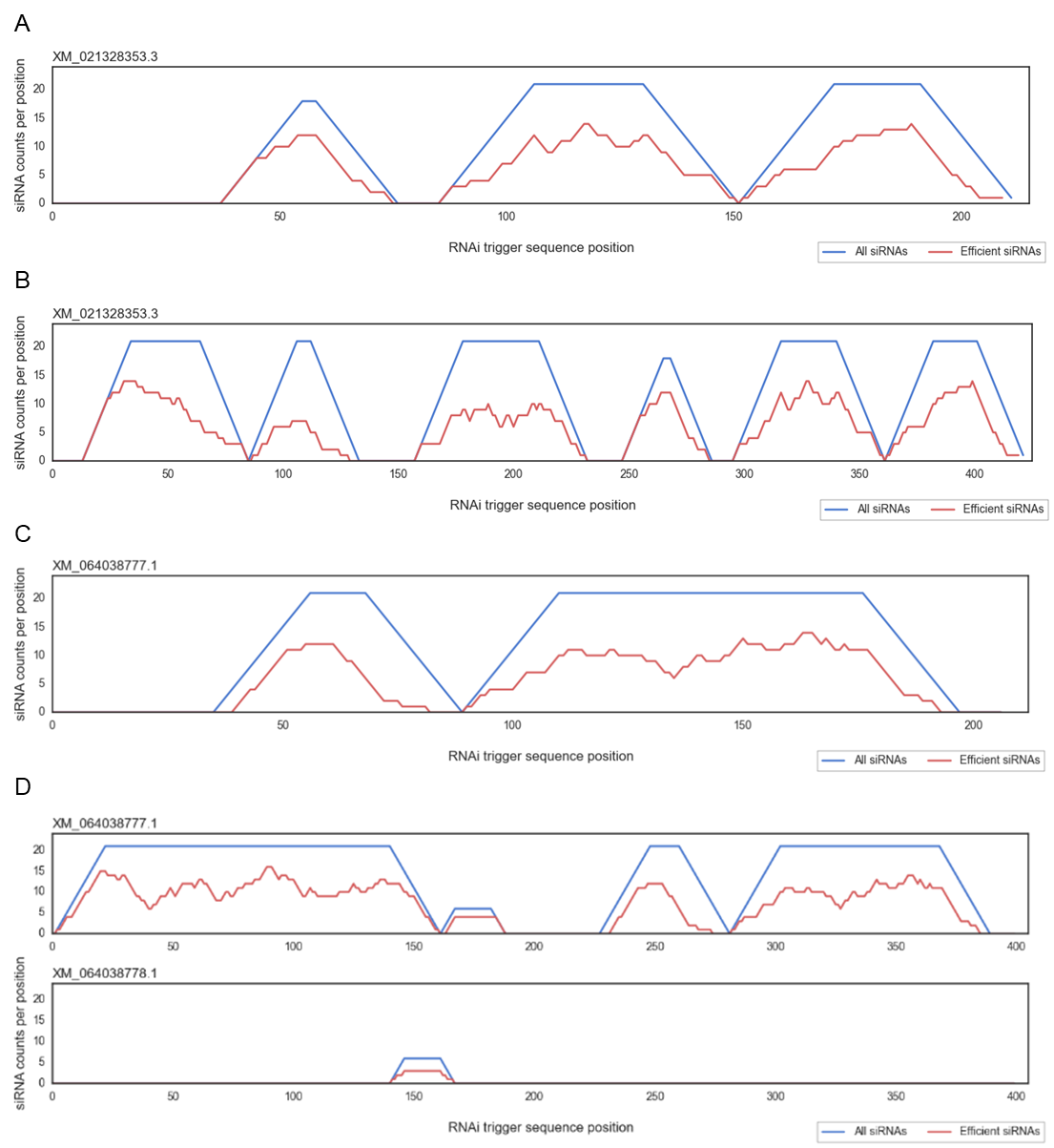

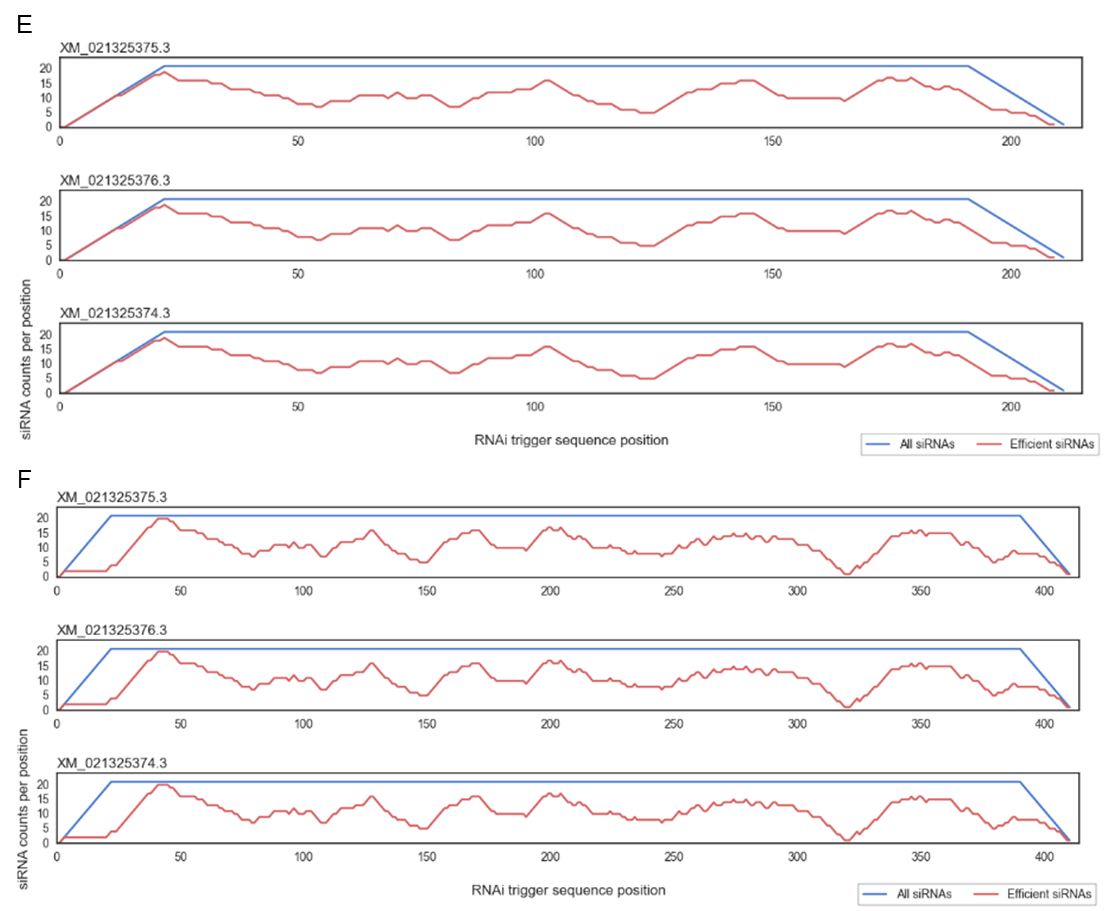

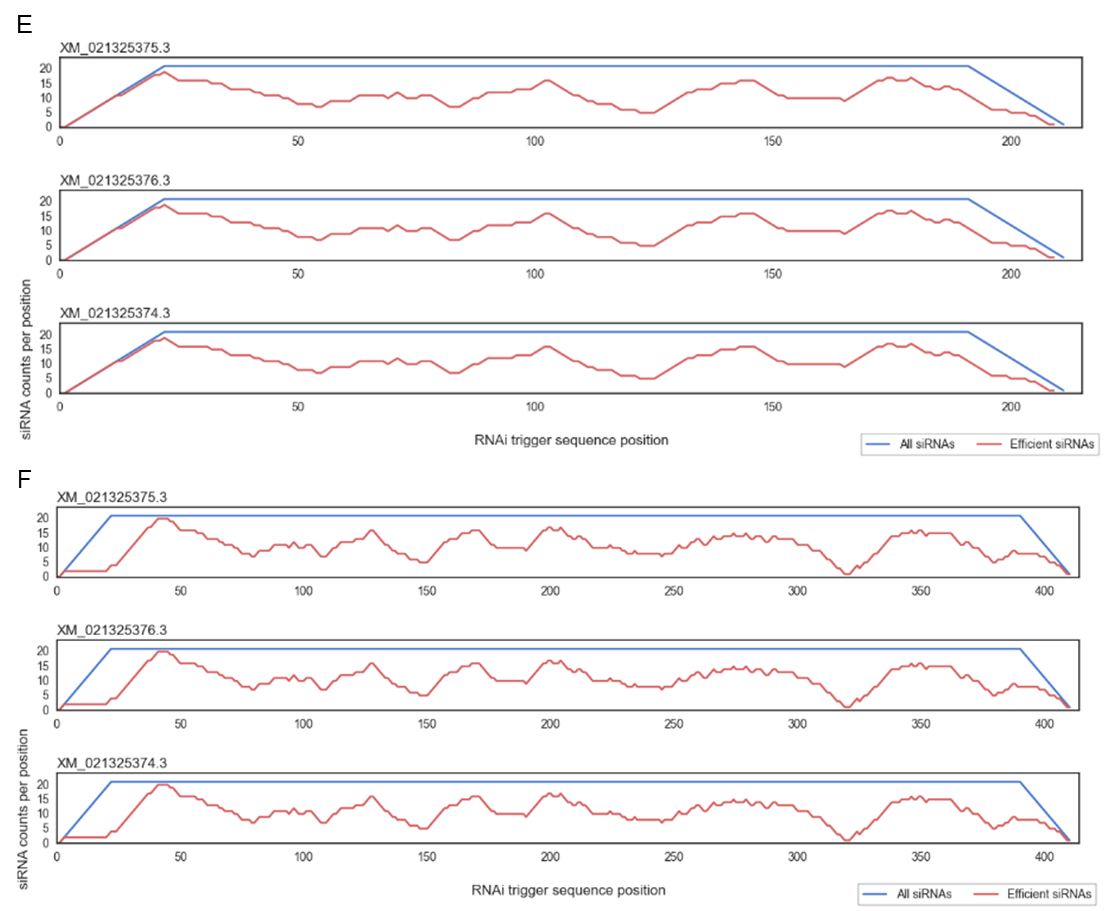


**Fig. S3**. Predicted siRNAs produced by our *in silico* designed dsRNAs against *H. armigera*. A) dsCHSII 420 bp. B) dsCHSII 210 bp. C) dsCYP6 400 bp. D) dsCHCYP6 207 bp. E) dsVATPa 409 bp. F) dsVATPa 210 bp. The algorithm assumes that Dicer can cleave the dsRNA molecule at any point. Each position presented in the X-axis indicates the possible start of a siRNA. Thus, the Y-axis indicates how many siRNAs derived from x starting point in the dsRNA sequence (x-mer) will have a hit in the mRNA target sequence given the parameters chosen.


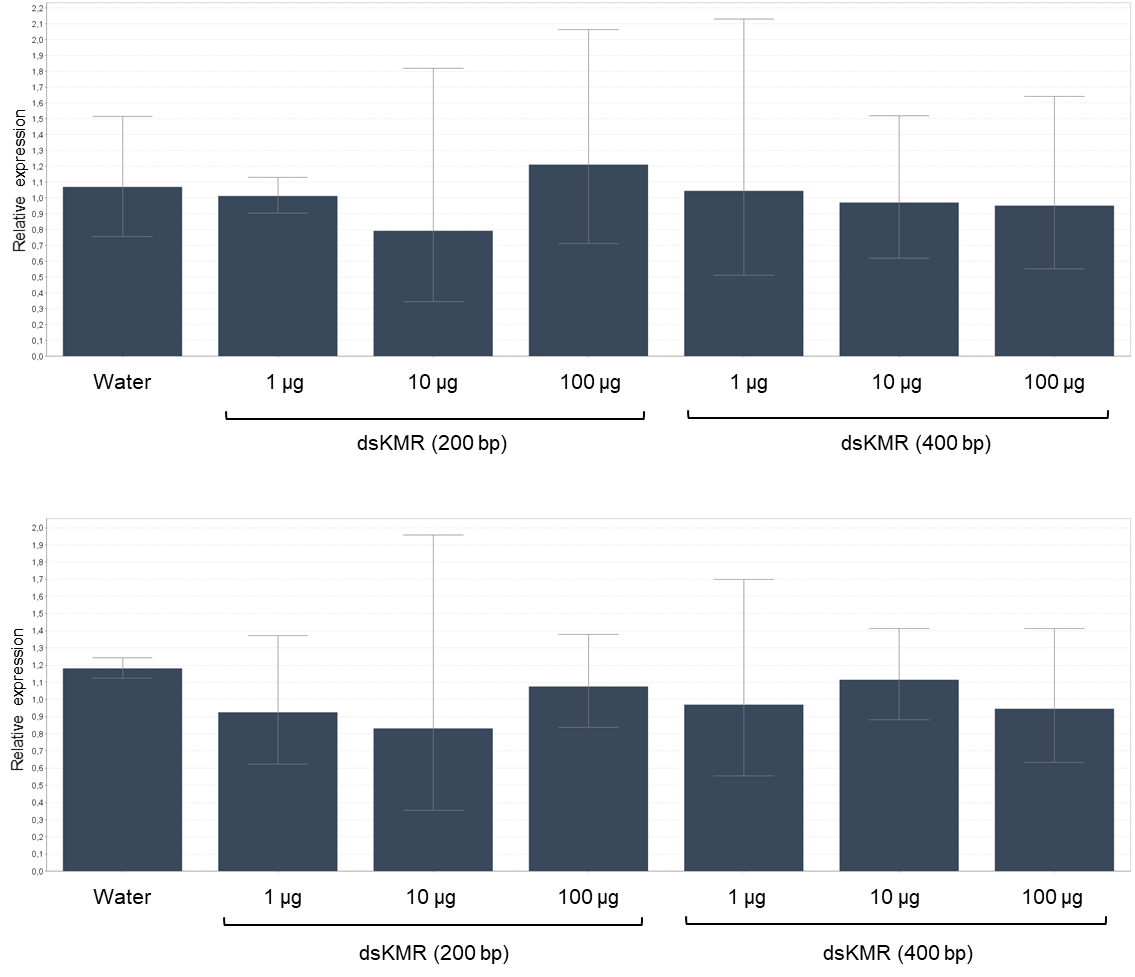


**Fig. S4.** Expression of *Ha*CHSII (top) and *Ha*CYP6 (bottom) after injection of control dsRNA molecules (dsKMR) with different sizes and amounts. Relative expression is presented as 2–∆∆Ct (fold-change values), with average expression as normalization factor. None statistical differences were observed under p-value < 0.05 (determined by one-way ANOVA). Error bars represent ±SE (n=9). Larvae were injected during the third instar stage. Samples were collected 48 hours post-injection. CHSII: chitin synthase II. CYP6: cytochrome P450 protein CYP6B6. KMR: kanamycin resistance gene.

**
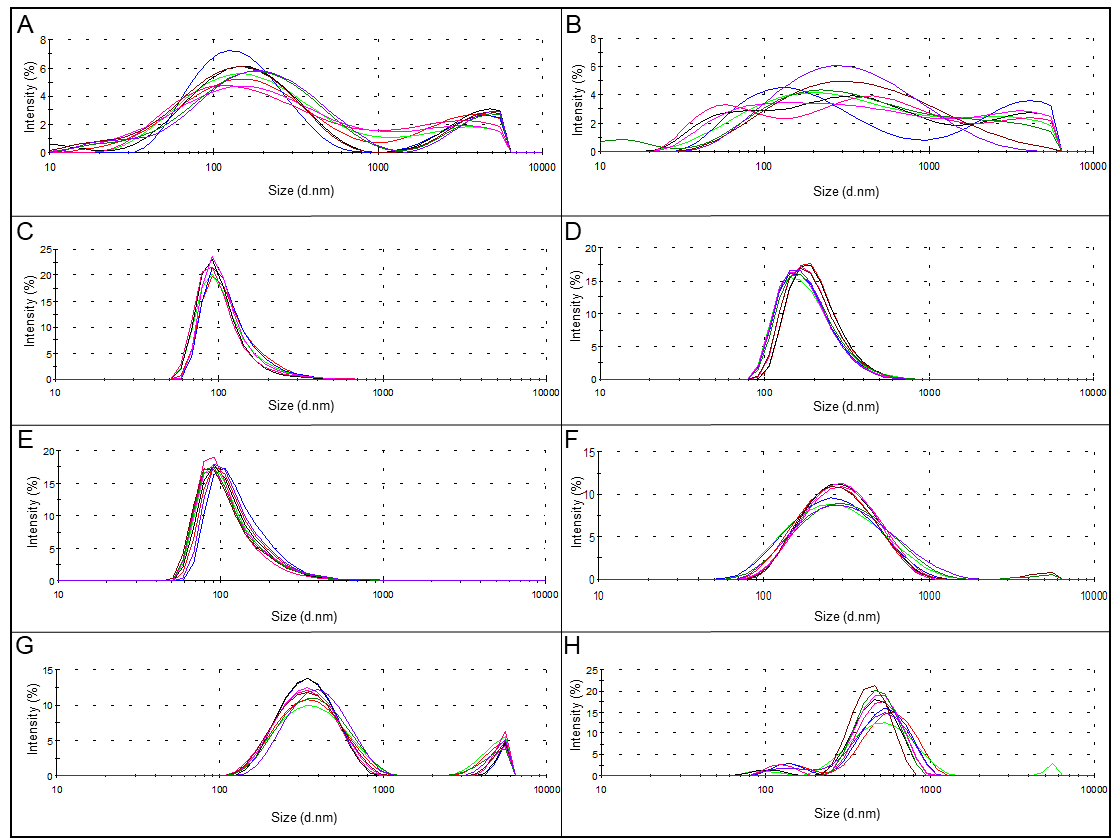
**

**Fig. S5**. Dynamic light scattering (DLS) analysis of dsRNA/CS/TPP nanoparticles. **A**) N:P=2:1, 200 bp. **B**) N:P=2:1, 400 bp. **C**) N:P=5:1, 200 bp. **D**) N:P=5:1, 400 bp. **E**) N:P=10:1, 200 bp. **F**) N:P=10:1, 400 bp. **G**) N:P=50:1, 200 bp. **H**) N:P=50:1, 400 bp. N:P indicates the phosporous:nitrogen ratio of the nanoparticle. Size of dsRNA fragment is given in bp.

**
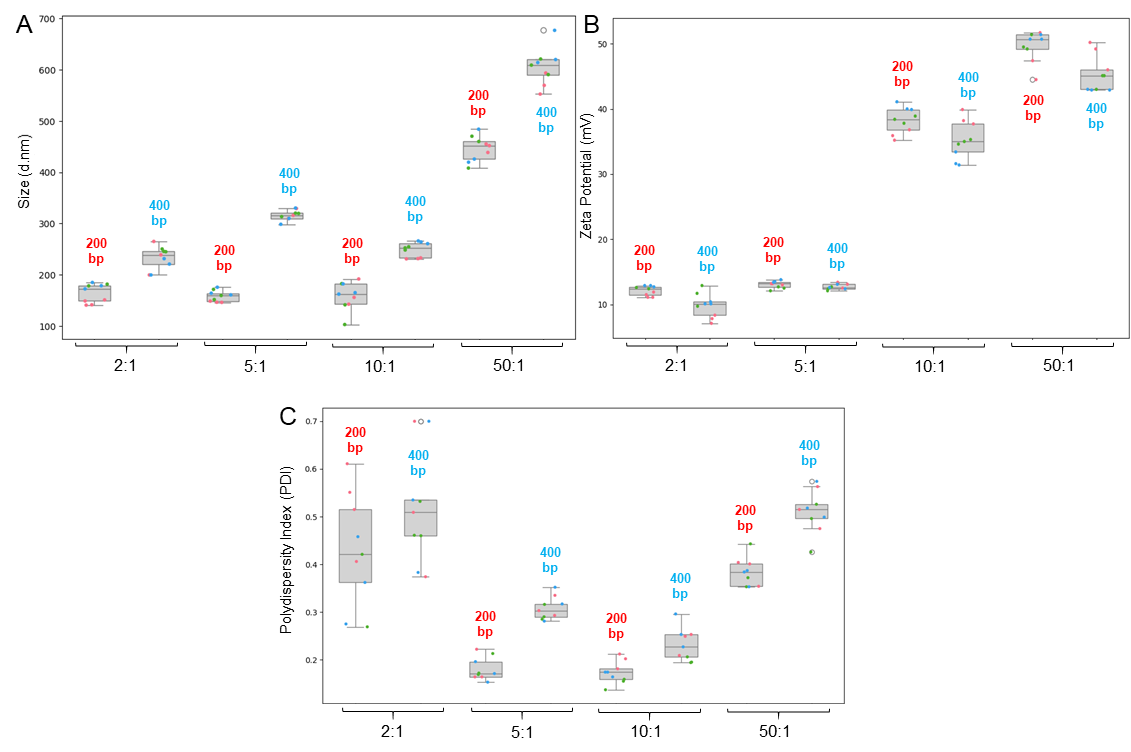
**

**Fig. S6**. Distribution of physicochemical parameters of chitosan/TPP/dsRNA nanoparticles measured by Dynamic Scattering Light Analysis**.** **A**) Size. **B**) Zeta potential. **C**) Polydispersity. X-axis indicates N:P ratio (phosporous:nitrogen) of the nanoparticle. Size of dsRNA fragment is given red and blue legends over the boxes.

**Table S1**. Artificial diet composition for *H. armigera* and *S. postica*.

| *Helicoverpa armigera* | |
| --- | --- |
| **Component** | **Amount (g or mL)** |
| White beans | 75 |
| Wheat germ | 60 |
| Soybean soluble protein | 30 |
| Lyophilized milk | 30 |
| Yeast | 37.5 |
| Agar | 30 |
| Ascorbic acid | 1.8 |
| Sorbic acid | 0.9 |
| Formaldehyde | 1.8 |
| Methylparaben | 1.5 |
| Distilled water | 1200 |
| *Scaptotrigona postica* | |
| **Component** | **For 1 mL % (w/v or v/v)** |
| Honey | 50% |
| Sucrose | 30% |
| Unflavored Gelatin | 2% |

**Table S2**. Primers used for RT-qPCR analyses. Melting temperature for all primers is 62 °C.

| **Gene** | **Primer Forward** 5' - 3' | **Primer Reverse** | **M-value** |
| --- | --- | --- | --- |
| CHSII | GTGCAATCTCAACAACGTATCG | TCATCACTCTTGCCGAACC | 0.62 |
| CYP6 | CAACTATACGCCCACATCTCG | CTGTGCCACTAAGAGATCATCG | 3.24 |
| VATPa | ACTCCGATGTCATTGTGTACG | GCATGTTGGATGTGTTGGC | 1.75 |
| RPS13 | CGGTGGTGTTTTTGTGACTCT | CTAACGATCCATCCGTGCGTATGC | 0.35 |
| RPS18 | GGTAGTGGTTGGCTTTCAACA | TGGAAAGTATGTCCCAAGAGC | 0.35 |

CHSII: chitin synthase II. CYP6: cytochrome P450 protein CYP6B6. VATPa: V-ATPase subunit a. RPS13 and RPS18: ribosomal proteins 13 and 18. M-values from geNorm stability analysis is showed.

**
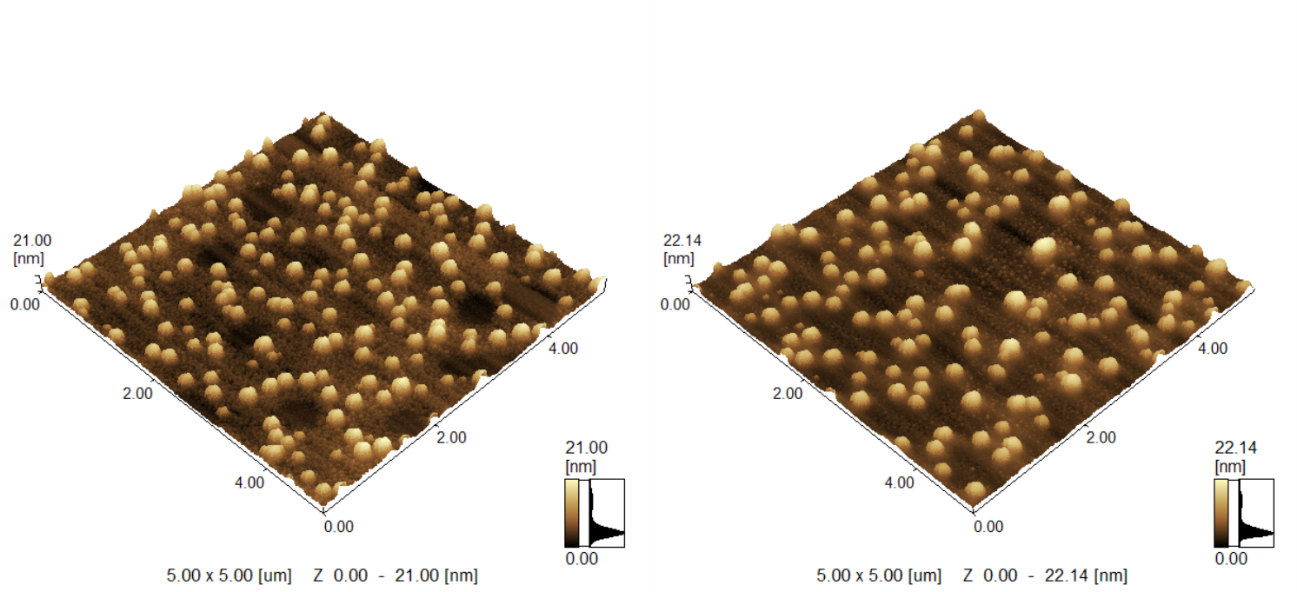
**

**Fig. S7**. Nanoparticles with 10:1 N:P ratio and a 400 bp-length dsRNA visualized by Atomic Force Microscopy (AFM) Analysis. Imaging was conducted in dynamic (tapping) phase mode using a rectangular cantilever with a conical silicon tip, featuring a nominal spring constant of 10–130 N/m and a resonance frequency range of 204–497 kHz. The sweep frequency was set to 1 Hz. Surface topography images were acquired over 5 µm × 5 µm areas with a resolution of 512 × 512 pixels.


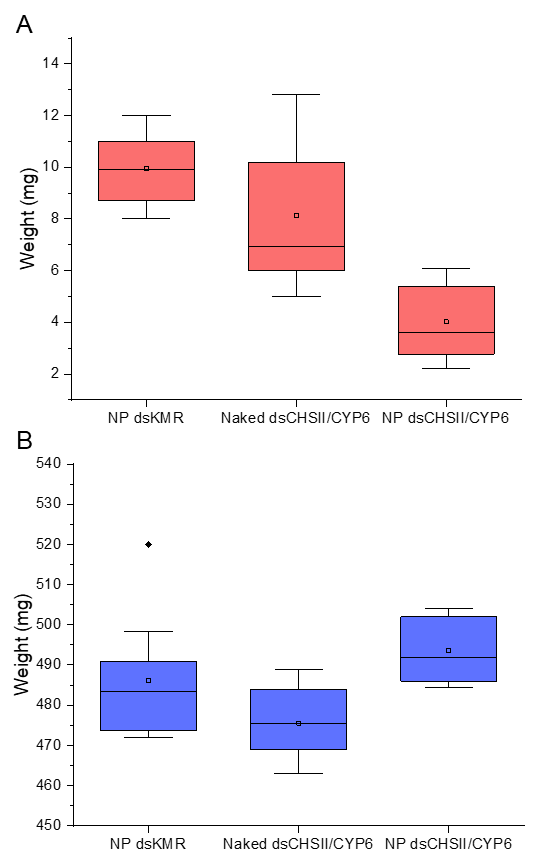


**Fig. S8**. Weight distribution of larvae fed with dsRNA molecules (encapsulated: NP and non-encapsulated: Naked). Red boxes indicate data from larvae at 4 DAI (A) and blue boxes data from larvae at 18 DAI (B). KMR: kanamycin resistance gene (mock control). CHSII: chitin synthase II. CYP6: cytochrome P450 6B6. DAI: days after ingestion of dsRNA.


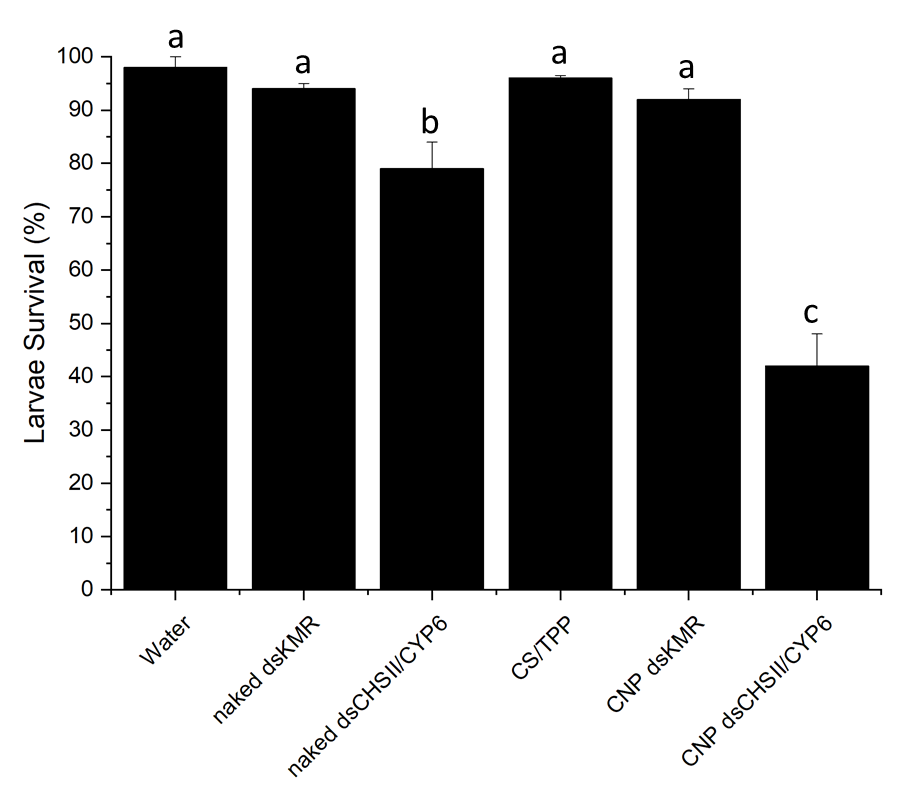


**Fig. S9**. *Helicoverpa armigera* larval survival after ingestion of diet containing different molecules. Neonate larvae were fed with diet containing (1µg/g) of naked dsRNA, CNPs, water or CS/TPP nanoparticles. Cumulative mortality after 35 days after ingestion was compared among treatments. Different lowercase letters indicate significant differences at a p-value < 0.05 (as determined by one-way ANOVA and Tukey’s HSD). **CHSII**: chitin synthase II. **CYP6**: cytochrome P450 protein CYP6B6. **KMR**: kanamycin resistance gene. **CNP**: chitosan/TPP/dsRNA nanoparticles. **CS/TPP**: nanoparticles of chitosan and TPP without dsRNA.
